# Erythropoietin resistance among pediatric patients on chronic hemodialysis: A cross-sectional study

**DOI:** 10.1007/s00467-025-06776-4

**Published:** 2025-04-28

**Authors:** Rasha Helmy, Fatina I. Fadel, Rasha E. Galal, Amira M. Mohammed, Shaimaa Sayed

**Affiliations:** Pediatric Department, Faculty of Medicine, Cairo University, 4 Extension of Nobar Street, Cairo, Egypt

**Keywords:** Erythropoietin resistance, Pediatrics, Kidney failure, Hemodialysis, Erythropoietin resistance index

## Abstract

**Background:**

The erythropoietin resistance index (ERI) is an accurate indicator of erythropoietin (EPO) resistance and is related to a worse prognosis in patients on hemodialysis (HD). ERI is simple, cheap and could be calculated easily in children receiving HD. We aimed to assess the EPO resistance in children with kidney failure on regular HD.

**Methods:**

An analytical cross-sectional study was conducted on 80 children with kidney failure on regular HD. They were assessed by history taking and laboratory investigations including complete blood count, C- reactive protein (CRP), iron, ferritin, parathyroid hormone and serum electrolytes. ERI was calculated.

**Results:**

The study included 80 patients; 41 (51.2%) were male. The mean age of the study group was 8.86 ± 2.76 years. Sixty-three patients (78.8%) were on iron therapy. Mean ERI was 28.87 ± 10.62. The ERI was significantly positively correlated with age (r = 0.242; *P = *0.031), EPO dose (r = 0.290; *P = *0.001) and CRP (r = 0.219; *P = *0.049). The ERI had a significantly negative correlation with KT/V (r = - 0.262; *P = *0.019), hemoglobin level (r = - 0.265; *P = *0.001) and platelet count (r = - 0.254; *P = *0.023).

**Conclusions:**

Erythropoietin resistance is associated with many risk factors, including high CRP and low KT/V. Inadequate HD is the most important risk factor for EPO resistance in children on chronic HD. Adequate HD is considered as a protective measure against EPO resistance.

**Graphical Abstract:**

**Figure:**
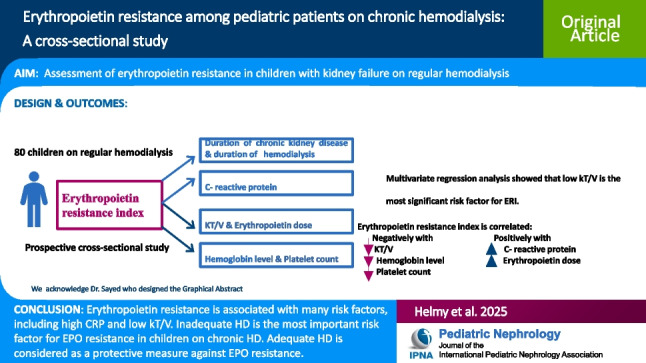
A higher resolution version of the Graphical abstract is available as Supplementary information

**Supplementary Information:**

The online version contains supplementary material available at 10.1007/s00467-025-06776-4.

## Background

Anemia is a common morbidity in children with chronic kidney disease (CKD). It is associated with increased mortality. It is caused by decreased production of erythropoietin (EPO) from the kidney. Also, immune activation that decreases iron absorption, EPO production and erythrocyte life span leading to iron-restricted erythropoiesis is called anemia of inflammation [1].

Anemia of CKD is treated using iron and erythropoiesis-stimulating agents (ESA) to stimulate erythropoiesis [2]. Most CKD patients with anemia are treated adequately using medium doses of these medications [3]. But higher requirements of ESA could be needed due to iron deficiency, hemodialysis (HD), malignancy, infection, inflammation, vitamin deficiencies, toxins, hyperparathyroidism and insufficient dialysis [4–6].

EPO resistance is defined as persistent decreased hemoglobin (Hb) level despite 3 months of treatment with high dose ESA. It is associated with inflammation and fluid retention [7]. It could be chronic or transient with circumstances such as infections or surgical intervention which could decrease the response to ESA [8].

Moreover, erythropoietin resistance index (ERI) is defined as average weekly EPO dose per kg body weight per average Hb [9]. ERI is an accurate indicator for EPO resistance and is associated with adverse effects in chronic HD [5]. Increased ERI may predict the risk of all-causes and cardiovascular mortality in HD patients [10].

Furthermore, the neutrophil-to-lymphocyte ratio (NLR) is an indicator for inflammation and endothelial dysfunction in patients with CKD [5]. It is easily calculated from complete blood count (CBC) and it is an important marker of inflammation in dialysis patients [11]. It is simple, cheap, and an interesting analytical parameter and could be routinely extracted in HD patients [12]. Moreover, the NLR role in EPO responsiveness remains unclear in HD patients [10]. In the current study we aimed to assess the EPO resistance in children with kidney failure on chronic HD.

## Methods

We conducted an analytical prospective cross-sectional study on 80 patients with kidney failure (CKD stage 5) treated by HD, at the Pediatric HD Unit of Cairo University. The study included children aged 1–14 years, of both sexes, who had been at least 3 months duration on regular HD. The exclusion criteria were hospital admission in the previous 3 months due to infection or malignancy, or a history of receiving a blood product transfusion in the last 3 months. After taking informed consent from the parents, all patients underwent the following procedures:

### History taking

We collected data about the underlying kidney disease, the duration of CKD and regularly used medications, including antihypertensive medication, iron and EPO supplementation.

### Laboratory data

Blood tests were conducted after HD sessions with the routine follow-up laboratory tests:

- Complete blood count: to assess Hb level, total leucocytic count, platelet count, lymphocytes count and neutrophil count. NLR was calculated.
- Serum iron, ferritin, C- reactive protein (CRP).
- Serum sodium, potassium, calcium, phosphorous, alkaline phosphatase (ALP) and parathyroid hormone (PTH).
- ERI was calculated through the following equation: recombinant human erythropoietin (rHuEPO) dose (units/week/kg)/Hb (gram/dl) and expressed as (IU/kg/w/g/dL).
- There is no gold standard reference range for ERI in children or adults. However, some studies use the mean value of ERI of their population to classify them into low ERI group (ERI ≤ mean value of ERI) and the high ERI group (ERI > mean value of ERI) based on the mean ERI value [13, 14].

### Sample size calculation

We aimed to assess the EPO resistance in children with kidney failure on chronic HD. Previous data indicate that standard deviation (SD) of control is 0.5 and SD of the regression error would be 1.7. If the true slope of the line obtained by regression patients against control is 2.5, we would need to assess 80 patients to reject the null hypothesis that this slope equals zero with probability (power) 95%. The type 1 error probability associated with this test of the null hypothesis is 0.05. Therefore, sample size was 80 patients.

### Statistical analysis

Patient data was processed by the Statistical Package for the Social Sciences (SPSS) version 28 (IBM Corp., Armonk, NY, USA). Data were summarized in the form of mean, SD, median, minimum and maximum in quantitative data and in the form of frequency (count) and relative frequency (percentage) for categorical data. Non-parametric Kruskal–Wallis and Mann–Whitney tests were used in comparisons between quantitative variables. Spearman correlation coefficient was used in correlations between quantitative variables. Linear regression analysis was done to detect independent predictors of ERI [15]. P-value less than 0.05 was considered as statistically significant.

## Results

The study included 80 patients who had kidney failure (stage 5 CKD) on regular HD and three patients were excluded due to history of receiving a blood product transfusion in the last 3 months. Forty-one of them were males (51.2%) and the other 39 were females (48.8%). The mean age of the children was 8.86 ± 2.76 years (range from 1–14 years). Their primary kidney disease is demonstrated in Table 1.

**Table 1:** Basic demographic and clinical data of the study group

	Mean ± SD	Range
Age (years)	8.86 ± 2.76	2.00–15.00
Duration of HD (months)	48.21 ± 5.06	12- 120
Duration of CKD (years)	6.14 ± 3.25	1.00–14.00
KT/V (liters/minute)	1.21 ± 0.06	1.10–1.30 l
EPO dose (IU/kg/week)	266.8 ± 72.1	98.7- 400.0
ERI (IU/kg/w/g/dL)	28.87 ± 10.62	7.3–47.8
	**Count**	**%**
Male (%)	41	51.2%
Female (%)	39	48.8%
Primary kidney disease		
Glomerular disease	20	25%
Ciliopathies	18	22.5%
Congenital anomalies of kidney and urinary tract	29	36.5%
Primary hyperoxaluria	10	12.5%
Alport syndrome	1	1.25%
Cystinosis	2	2.5%

*SD* standard deviation, *HD* hemodialysis, *CKD* chronic kidney disease, *KT/V* a measure of dialysis adequacy, *EPO* erythropoietin, *ERI* erythropoietin resistance index

The patients were receiving regular HD three times per week, with the exception of one child who received dialysis four times weekly. Duration of HD was 48.21 ± 5.06 months ranging between 12 and 120 months. Mean KT/V was 1.21 ± 0.06 L/minute ranging between 1.10 and 1.30 L/minute. All children in the current study received subcutaneous recombinant human erythropoietin injection (Epoetin alfa) with a mean dose 266.8 ± 72.1 IU/kg/week ranging between 98.7 and 400.0 IU/kg/week. Sixty-three patients (78.8%) were receiving iron therapy and 59 patients (73.7%) had anemia. Fifty-four patients (67.5%) were hypertensive on amlodipine and captopril as antihypertensive medications. Duration of CKD was 6.14 ± 3.25 years (1.00–14.00) (Table 1).

The laboratory data are demonstrated in Supplementary Table 1. The mean of the NLR level was 1.78 ± 1.27 ranging between 0.15 and 7.9. Mean CRP level was 10.8 ± 9.5 mg/L ranging between 6.0 and 48.0 mg/L. Mean ERI was 28.87 ± 10.62 (IU/kg/w/g/dL) ranging between 7.3 and 47.8. We classified the study population according to mean value of ERI into 2 groups: low ERI group that included 42 patients with mean ERI 20 ± 4.7 (IU/kg/w/g/dL) ranging from 7.3 to 28 and high ERI group that included 38 patients with mean ERI 38.6 ± 5.3 (IU/kg/w/g/dL) ranging from 29.1 to 47.8. Relations between ERI with clinical data and primary kidney disease are demonstrated in Table 2.

**Table 2:** Relation between erythropoietin resistance index and clinical data and primary kidney disease

	Erythropoietin Resistance Index (IU/kg/w/g/dL)	
	Mean ± SD (Range)	*P* value
Male	28.41 ± 11.26 (7.3 − 47.10)	0.75
Female	29.34 ± 10.01 (13.70–47.80)	
Receiving iron therapy	29.16 ± 10.47 (7.3 − 47.10)	0.62
Not receiving iron therapy	27.76 ± 10.37 (14.00–44.80)	
Receiving anti-hypertensive drug	28.43 ± 11.01 (7.3 − 47.80)	0.56
Not receiving anti-hypertensive drug	29.76 ± 9.90 (13.9 − 47.10)	
Primary kidney disease		
Glomerular disease	32.75 ± 11.78 (13.70 − 46.50)	0.018*
Ciliopathies	21.37 ± 8.22 (7.3 − 42.90)	
Congenital anomalies of kidney and urinary tract	31.40 ± 8.94 (17.90 − 47.80)	
Primary hyperoxaluria	30.30 ± 9.61 (14.30 − 47.10)	
Cystinosis	26.90 ± 10.93 (12.10 − 41.70)	

*SD* standard deviation

ERI significantly positively correlated with age (r = 0.24; *P = *0.031) and EPO dose (r = 0.29; *P = *0.05). ERI significantly negatively correlated with KT/V (r = − 0.262; *P = *0.019), while the correlation was not significant between ERI and gender, iron therapy, anti-hypertensive treatment or duration of HD. There was significant difference between different kidney disease categories regarding ERI (*P = *0.018) (Table 3).

**Table 3:** Correlation between erythropoietin resistance index with clinical and laboratory investigations

	Erythropoietin Resistance Index	
	Correlation Coefficient	*P* value
Age (Year)	0.24	0.031*
KT/V (liters/minute)	− 0.262	0.019*
Dose erythropoietin (IU/Kg/Week)	0.29	**0.05**
Duration of chronic kidney disease (years)	0.176	0.119
Duration of HD (months)	0.166	0.12
Hemoglobin (g/dl)	− 0.265	**0.001**
Total leucocyte count (10^/cmm)	− 0.093	0.413
Platelet count (10^/cmm)	− 0.254	**0.023**
Lymphocyte (%)	− 0.095	0.402
Neutrophil (%)	0.095	0.401
Neutrophil Lymphocyte Ratio	0.126	0.267
C-reactive protein (mg/L)	0.219	**0.049**
Sodium (mmol/l)	− 0.017	0.881
Potassium (mmol/l)	0.079	0.486
Iron (ug/dl)	0.011	0.925
Ferritin (ng/ml)	0.079	0.486
Total Calcium (mg/dl)	0.205	0.068
Phosphorus (mg/dl)	0.120	0.290
Parathyroid hormone (pg/ml)	0.115	0.311
Alkaline phosphatse (U/L)	0.154	0.174

*HD* hemodialysis, *KT/V* a measure of dialysis adequacy

**Fig. 1:**
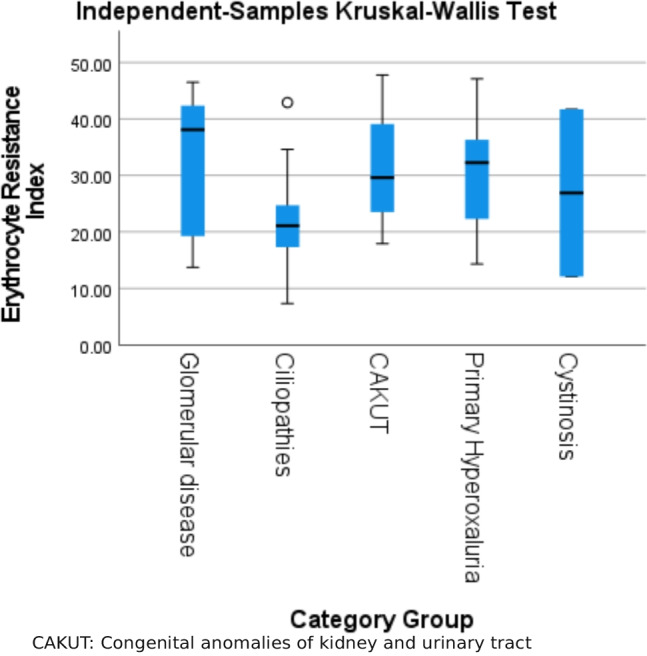
Post hoc comparison of different primary kidney disease categories regarding erythropoietin resistance index

Post hoc comparison revealed significantly higher ERI in patients with congenital anomalies of the kidney and urinary tract, glomerular diseases or tubular diseases than ERI in patients with ciliopathies (Fig. 1). ERI had significant negative correlation with Hb levels (r = − 0.265; *P = *0.001) and platelet count (r = − 0.254; *P = *0.023), while ERI had positive correlation with CRP levels (r = 0.219; *P = *0.049). Otherwise, there was no significant correlation between ERI and other laboratory investigations. There was positive non-significant correlation between ERI and NLR (Table 3). There was no significant correlation between NLR and Hb, iron, ferritin levels or dose of EPO (Table 4). Multivariate regression analysis for risk factors for EPO resistance (age, duration of CKD, CRP and KT/V) showed that inadequate dialysis (low KT/V) is the most important risk factor for EPO resistance (high ERI) with p-value = 0.020 (Table 5).

**Table 4:** Correlations between neutrophil lymphocyte ratio and hemoglobin, iron, ferritin levels and dose of erythropoietin

	NLR	
	Correlation Coefficient	*P* value
Hemoglobin (g/dl)	0.130	0.250
Iron (ug/dl)	− 0.115	0.310
Ferritin (ng/ml)	0.043	0.706
Dose of erythropoietin (IU/Kg/Week)	0.191	0.091

*NLR* neutrophil lymphocyte ratio

**Table 5:** Multivariate regression analysis for risk factors of erythropoietin resistance index

Model		Unstandardized Coefficients		Standardized Coefficients	t	*P* value	95.0% Confidence Interval for B	
		B	Std. Error	Beta			Lower Bound	Upper Bound
ERI	(Constant)	81.601	22.207		3.675	< 0.001	37.391	125.811
	Age (Year)	2.530	6.538	0.020	0.387	0.700	− 10.500	15.559
	Duration of chronic kidney disease (Years)	− 0.768	5.596	− 0.007	− 0.137	0.891	− 11.920	10.384
	C-reactive protein (mg/L)	0.948	1.233	0.026	0.769	0.445	− 1.510	3.405
	KT/V	− 43.674	18.366	− 0.260	− 2.378	0.020	− 80.237	− 7.110

*ERI* erythrocyte resistance index, *KT/V* a measure of dialysis adequacy

## Discussion

Anemia in patients with CKD is caused mainly by deficient EPO production [16]. The management of anemia with ESA and iron is the main treatment of patients on HD [17]. ERI is an indicator of EPO resistance and is associated with many risk factors [5]. Poor response to EPO in the pediatric population is affected by clinical characteristics such as chronic inflammation, inadequate dialysis, hyperparathyroidism, and blood loss rather than chronological age. In contrast, due to inadequate supplementation, iron and folate deficiencies are the major causes in adults [18]. The aim of the current study was to identify the EPO resistance in children with kidney failure on chronic HD.

In the current study, the mean ERI was 28.87 ± 10.62. This mean value is different and relatively higher than other populations. Pınarbaşı et al. found that ERI value was 13.01 ± 7.52 U/kg/week/g/dL in children receiving peritoneal dialysis (PD) as a modality for kidney replacement therapy [19]. PD patients have lower ESA requirements and a decreased ERI than HD patients [20]. Moreover, the loss of blood that occurs during HD sessions could cause the increased EPO requirements in HD patients [21].

Also, Kaya et al. reported that the mean ERI value was 15.7 IU/kg/w/g/dL in their study that included 33 children with kidney failure, 20 of whom were receiving HD and 13 of whom were receiving PD [13]. The differences between the Kaya et al. study and this current study could be multifactorial and include differences in EPO formulation, type of dialysis, and study size. In the Kaya et al. study some children were receiving darbepoetin alfa which has a longer half-life that maintains Hb levels in a more stable fashion and reduces ERI levels more effectively than epoetin alfa [22, 23]. But all children included in the current study were receiving epoetin alfa. Another difference is that the Kaya et al. study included children receiving PD who had lower ESA requirements [20]. All children included in the current study were on chronic HD. Lastly, the Kaya et al. study included 33 children which is a relatively small sample size in comparison with the current study that included 80 children.

Some studies use the mean value of ERI of their population to classify them into a low ERI group (ERI ≤ mean value of ERI) and a high ERI group (ERI > mean value of ERI) based on the mean ERI value [13, 14]. A study that included 1270 adult patients with CKD divided the patients into low ERI and high ERI, according to the median ERI, which was 14.03. The median value in the low group was 9.2 (6.4–11.6) and 19.4 (16.4–25.7) in the high group [14]. Moreover, the relationship between age and EPO resistance was confounded by a disproportionate prescription of larger EPO doses for the pediatric population than for the adults [18].

In the current study, 41 (51.2%) patients were male. No significant correlation was reported between ERI and gender. The effect of gender on ESA response is debatable. A previous study conducted on an adult population found that females had significantly higher ERI compared to male HD patients, which is in agreement with other publications identifying the relation between gender and EPO requirements. The authors attributed their finding to the fact that females required higher doses of EPO to reach the target Hb than males. So, it might not indicate a difference in responsiveness to EPO [24]. Moreover, Chung et al. found that females had higher resistance to EPO treatment [25].

The mean age of the children in the current study was 8.86 ± 2.76 years. ERI positively correlated with age. In disagreement with our finding, a total of 100 children on PD were included in a study conducted on children less than 5 years of age who had very high ERI values. The difference can be attributed to the different population types in both studies (children with PD versus HD) [19]. Another study reported that PD patients showed a decreased EPO requirement than HD patients, to achieve the same Hb level [20].

In the current study, no significant correlation was reported between ERI and the anti-hypertensive medications amlodipine and angiotensin converting enzyme inhibitors (ACEI). In discordance with our study, the use of iron and high dosage of ACEI/angiotensin II-receptor blockers were protective factors against EPO resistance among CKD patients in a study conducted on 1080 ESA users [26]. The difference could be attributed to inclusion of all CKD patients in the previous study, not only HD patients. There are debatable opinions regarding ACEI use and EPO resistance [19, 27, 28]. It has been reported that ACEI decrease dose-dependent EPO synthesis [29].

In the current study, no significant correlation was reported between ERI and iron therapy. In contrast with our finding, concomitant use of iron was protective against EPO resistance among CKD patients in a study by Ingrasciotta et al., which was conducted on 1080 ESA users [26]. The difference can be attributed to inclusion of all CKD patients in the previous study, not only HD patients. Also, they excluded blood transfusion only 1 month before the study while we excluded patients who received blood transfusion in the last 3 months.

The follow-up of the ERI may be helpful in the early detection of the EPO resistance in patients on chronic HD. It helps to avoid high dose of EPO therapy, which is associated with reduced survival [24]. Mean EPO dose in the current study was 266.8 ± 72.1 IU/kg/week ranging between 98.7 and 400.0 IU/kg/week. ERI positively correlated with EPO dose. In line with our finding, the actual EPO doses in the Chung et al. study were significantly elevated in patients with higher ERI [25].

Mean Hb among the study population was 10.44 ± 2.00 g/dL ranging between 5.20 and 16.10 g/dL. ERI in the present study was found to be negatively correlated with Hb levels. In contrast to our study, Zhang et al. studied 299 HD patients and found that the EPO responsiveness was negatively related to Hb [10]. Moreover, Valga et al. reported that ERI is correlated positively with Hb level [30]. The differences could be attributed to larger population size and inclusion of adult patients in the previous two studies [10, 30]. However, Chait et al. demonstrated that ESA affect ERI more significantly than Hb because the doses are reportedly 9- to 13-fold more variable than Hb [31].

Despite the significant correlations between ERI with Hb level and EPO dose, both low Hb level and high EPO dose are not considered risk factors for high ERI as both of them are included in the equation which we use in ERI calculation. So, we excluded them from the multivariate regression analysis in the current study to avoid mathematical instability.

Mean platelet count was 245.2 ± 101.6 10^/cm ranging between 52 and 626. ERI was found to be negatively correlated with platelet count. In agreement with our results, another study reported that HD patients with a higher concentration of platelets respond to EPO therapy better than those with a lower concentration [32]; which means that low platelet count is associated with hypo-responsiveness to ESA. Conversely, Valga et al. reported a positive correlation between ERI and platelet count [30]. The differences can be attributed to inclusion of adult patients in the Valga et al. study.

Mean CRP level in the present study was 10.80 ± 9.52 mg/I ranging between 6 and 48 mg/I. ERI positively correlated with serum CRP levels. This agreed with a study that reported ERI positively correlated with CRP [30]. Moreover, another study reported that HD patients with positive CRP had increased ERI compared to patients with negative CRP [24]. Furthermore, Kimachi et al. found that high levels of CRP in HD patients were associated with EPO resistance [33].

In the current study, mean NLR was 1.78 ± 1.27 ranging between 0.15 and 7.90. A positive non-significant correlation was reported between ERI and NLR. (r = 0.126; *P = *0.267). In agreement with our study, high NLR in the Pineault et al. study was associated with a non-significant elevated ERI [11], while there was no association between NLR and ERI in the Taymez et al. study [5].

In disagreement with our finding, a multivariate linear regression in the Zhang et al. study revealed that NLR was correlated with higher ERI [14]. Moreover, Valga et al. reported that NLR is positively correlated with ERI [30]. The difference in significance between the studies can be attributed to their larger sample sizes [10, 30]. Up to 10% of CKD patients have EPO resistance. Risk factors of EPO resistance are not all well-known, but relationship with inflammation has been identified [34]. In our study, patients with infections were excluded.

In the current study, mean ferritin level was 805.93 ± 578.95 g/dl ranging between 96.00 and 3507.00 g/dl. ERI was found to be positively correlated with serum ferritin levels. Yet, the correlation was non-significant. In concordance with our results, Joksimovic Jovic et al. revealed that patients who had EPO resistance had significantly higher serum ferritin levels than patients without EPO resistance [35]. Also, Valga et al. found a non-significant correlation between ERI and ferritin [30]. Another study reported a significant positive correlation between ERI and ferritin level [36].

The pathophysiological stress that increases the cortisol level decreases the levels of lymphocytes. This may lead to impaired immune response which causes EPO resistance [37]. In the current study, mean lymphocyte percentage was 33.06 ± 9.98% ranging between 10.00 and 66.00% and ERI was found to have non-significant correlation with lymphocytic count. This disagreed with Zhang et al. who found that the EPO responsiveness was negatively related to serum lymphocytes [10].

Multivariate regression analysis in the current study showed that low KT/V is the most significant risk factor for ERI. Inadequate dialysis can cause ESA resistance [17]. ERI in the current study was found to be negatively correlated with KT/V (which is a marker of dialysis adequacy). Our study is In agreement with a study conducted in adult patients that reported a relationship between ESA resistance and KT/V [24]. Also, there is a relationship between KT/V and ERI in HD patients [28]. The duration of HD session has effect on EPO response; in a study conducted by Maeda et al. that included 300 HD patients, the addition of 1 h of HD could decrease the EPO dose by approximately 2000 IU/week [38].

## Conclusions

EPO resistance is associated with many risk factors, including high CRP and low KT/V. Inadequate HD is the most important risk factor for EPO resistance. Hence, adequate hemodialysis is considered as a protective measure against EPO resistance. Low Hb level and high EPO dose are considered predictors of EPO resistance. The current study is one of the largest single center studies evaluating EPO resistance in pediatric patients with kidney failure on chronic HD. Another strength of the current study is the assessment of different risk factors of EPO resistance in these patients.

## Limitations

The study is a single center study and more prospective multi-centric studies on larger scales are required to evaluate the EPO resistance in pediatric patients with kidney failure on chronic HD. Another limitation is the lack of a “gold standard” reference range for ERI in the pediatric population. Also, it is a cross-sectional study including a single evaluation for ERI and risk factors of EPO resistance. Longitudinal studies that include multiple assessments for EPO resistance with trial of correction of modifiable risk factors such as improvement of HD adequacy with higher KT/V could be informative.

## Supplementary Information

Below is the link to the electronic supplementary material.

Graphical abstract (PPTX 36 KB)

Supplementary file2 (DOCX 16 KB)

## Data Availability

All data generated or analyzed during this study are included in this published article.

## References

[1] Weiss G, Ganz T, Goodnough LT (2019) Anemia of inflammation. Blood 133:40–50 30401705 10.1182/blood-2018-06-856500 PMC6536698

[2] Portoles J, Martin L, Broseta JJ, Cases A (2021) Anemia in chronic kidney disease: from pathophysiology and current treatments to future agents. Front Med 8:642296 10.3389/fmed.2021.642296 PMC8032930 33842503

[3] Birnie K, Caskey F, Ben-Shlomo Y, Sterne JA, Gilg J, Nitsch D et al (2017) Erythropoiesis-stimulating agent dosing, haemoglobin and ferritin levels in UK haemodialysis patients 2005–13. Nephrol Dial Transplant 32:692–698 27190350 10.1093/ndt/gfw043 PMC5410985

[4] Putri RGP, Sholihah A, Sukirto NW (2020) Risk factor for erythropoietin resistance in hemodialysis patient: literature review. Ahmad Dahlan Med J 1:33–49

[5] Taymez DG, Ucar E, Turkmen K, Ucar R, Afsar B, Gaipov A et al (2016) The predictive value of platelet/lymphocyte ratio in hemodialysis patients with erythropoietin resistance. Ther Apher Dial 20:118–121 26929254 10.1111/1744-9987.12380

[6] Eriguchi R, Taniguchi M, Ninomiya T, Hirakata H, Fujimi S, Tsuruya K et al (2015) Hyporesponsiveness to erythropoiesis-stimulating agent as a prognostic factor in Japanese hemodialysis patients: the Q-Cohort study. J Nephrol 28:217–225 25080399 10.1007/s40620-014-0121-9

[7] Weir MR (2021) Managing anemia across the stages of kidney disease in those hyporesponsive to erythropoiesis-stimulating agents. Am J Nephrol 52:450–466 34280923 10.1159/000516901

[8] McMurray J, Parfrey P, Adamson JW, Aljama P, Berns JS, Bohlius J et al (2012) Kidney disease: Improving global outcomes (KDIGO) anemia work group. KDIGO clinical practice guideline for anemia in chronic kidney disease. Kidney Int Suppl 2:279–333

[9] Fujikawa T, Ikeda Y, Fukuhara S et al (2012) Time-dependent resistance to erythropoiesis-stimulating agent and mortality in hemodialysis patients in the Japan dialysis outcomes and practice patterns study. Nephron Clin Pract 122:24–32 23486237 10.1159/000346740

[10] Zhang J, Lu X, Wang S, Li H (2022) Neutrophil-to-lymphocyte ratio and erythropoietin resistance among maintenance hemodialysis patients. Blood Purif 51:708–713 34649238 10.1159/000519644

[11] Pineault J, Lamarche C, Bell R, Lafrance JP, Ouellet G, Leblanc M et al (2017) Association of neutrophil-to-lymphocyte ratio with inflammation and erythropoietin resistance in chronic dialysis patients. Can J Kidney Health Dis 4:2054358117735563 29147572 10.1177/2054358117735563 PMC5673002

[12] Martínez-Urbistondo D, Beltrán A, Beloqui O, Huerta A (2016) The neutrophil–lymphocyte ratio as a marker of systemic endothelial dysfunction in asymptomatic subjects. Nefrologia 36:397–403 26923388 10.1016/j.nefro.2015.10.018

[13] Kaya M, Cicek N, Guven S, Alpay H, Gokce I (2024) Resistance to erythropoiesis stimulating agents in children receiving renal replacement therapy. Clin Pediatr (Phila). 10.1177/00099228241299893 39690475 10.1177/00099228241299893

[14] Brooks A (2024) Erythropoietin resistance increases risk of mortality in patients with anemia, CKD. https://www.hcplive.com/view/erythropoietin-resistance-increases-risk-mortality-patients-with-anemia-ckd. Accessed 10 March 2025

[15] Chan YH (2004) Biostatistics 201: linear regression analysis. Singapore Med J 45:55–61 14985842

[16] Hazin MAA (2020) Anemia in chronic kidney disease. Rev Assoc Med Bras 66:s55–s58 10.1590/1806-9282.66.S1.55 31939536

[17] Rivera RF, Alibrandi MTS, Di Lullo L, Fioccari F (2017) Clinical management of anemia in patients with CKD. G Ital Nefrol 34(Suppl 69):20–35 28682026

[18] Bamgbola OF, Fredrick J, Kaskel FJ, Coco M (2009) Analyses of age, gender and other risk factors of erythropoietin resistance in pediatric and adult dialysis cohorts. Pediatr Nephrol 24:571–579 18800231 10.1007/s00467-008-0954-3

[19] Pınarbaşı AS, Dursun I, Günay N, Baatar B, Yel S, Dursun J et al (2021) Erythropoietin resistance index and the affecting factors in children with peritoneal dialysis. Blood Purif 50:942–951 33784664 10.1159/000514060

[20] Coronel F, Herrero JA, Montenegro J, Fernandez C, Gandara A, Conesa J et al (2003) Erythropoietin requirements: a comparative multicenter study between peritoneal dialysis and hemodialysis. J Nephrol 16:697–707 14733416

[21] Duong U, Kalantar-Zadeh K, Molnar MZ et al (2012) Mortality associated with dose response of erythropoiesis-stimulating agents in hemodialysis versus peritoneal dialysis patients. Am J Nephrol 35:198–208 22286821 10.1159/000335685 PMC3326284

[22] Macdougall IC (2002) Darbepoetin alfa: a new therapeutic agent for renal anemia. Kidney Int Suppl 80:55–61 10.1046/j.1523-1755.61.s80.11.x 11982814

[23] Arrieta J, Moina I, Molina J et al (2014) Switch from epoetin to darbepoetin alfa in hemodialysis: dose equivalence and hemoglobin stability. Int J Nephrol Renovasc Dis 7:353–359 25336984 10.2147/IJNRD.S61895 PMC4199978

[24] Osman A, Alkareem NA, EldinElawad B, Dawod O, Elshiekh M (2021) Impact of gender, C-reactive protein and body mass index on erythropoietin resistance index in maintenance hemodialysis patients. J Renal Endocrin 7:e04

[25] Chung S, Song HC, Shin SJ, Ihm SH, Park CS, Kim HY et al (2012) Relationship between erythropoietin resistance index and left ventricular mass and function and cardiovascular events in patients on chronic hemodialysis. Hemodial Int 16:181–187 22103889 10.1111/j.1542-4758.2011.00644.x

[26] Ingrasciotta Y, Lacava V, Marcianò I, Giorgianni F, Tripepi G, D’Arrigo G et al (2019) In search of potential predictors of erythropoiesis-stimulating agents (ESAs) hyporesponsiveness: a population-based study. BMC Nephrol 20:359 10.1186/s12882-019-1554-0 PMC6744676 31521117

[27] Zadrazil J, Horak P (2015) Pathophysiology of anemia in chronic kidney diseases: a review. Biomed Pap Med Fac Univ Palacky Olomouc Czech Repub 159:197–202 24401900 10.5507/bp.2013.093

[28] Okazaki M, Komatsu M, Kawaguchi H, Tsuchiya K, Nitta K (2014) Erythropoietin resistance index and the all-cause mortality of chronic hemodialysis patients. Blood Purif 37:106–112 24603656 10.1159/000358215

[29] Ryta A, Chmielewski M, Debska-Slizien A, Jagodzinski P, Sikorska-Wisniewska M, Lichodziejewska-Niemierko M (2017) Impact of gender and dialysis adequacy on anaemia in peritoneal dialysis. Int Urol Nephrol 49:903–908 28058668 10.1007/s11255-016-1499-1 PMC5403856

[30] Valga F, Monzón T, Henriquez F, Santana-del-Pino A, Antón-Pérez G (2020) Platelet-to-lymphocyte and neutrophil-to-lymphocyte ratios as markers of erythropoietin resistance in chronic haemodialysis patients: a multicentre cross-sectional study. Nefrologia 40:320–327 31839207 10.1016/j.nefro.2019.09.007

[31] Chait Y, Kalim S, Horowitz J, Hollot CV, Ankers ED, Germain MJ et al (2016) The greatly misunderstood erythropoietin resistance index and the case for a new responsiveness measure. Hemodial Int 20:392–398 26843352 10.1111/hdi.12407 PMC4934130

[32] Rėkutė S (2017) Influence of blood inflammatory parameters to erythropoietin resistance in haemodialysis patients. Porto Biomed J 2:176–246 10.1016/j.pbj.2017.07.053 PMC6806931 32258665

[33] Kimachi M, Fukuma S, Yamazaki S, Yamamoto Y, Akizawa T, Akiba T et al (2015) Minor elevation in C-reactive protein levels predicts incidence of erythropoiesis-stimulating agent hyporesponsiveness among hemodialysis patients. Nephron 131:123–130 26344924 10.1159/000438870

[34] Johnson DW, Pollock CA, Macdougall IC (2007) Erythropoiesis-stimulating agent hyporesponsiveness. Nephrology 12:321–330 17635745 10.1111/j.1440-1797.2007.00810.x

[35] JoksimovicJovic J, Antic S, Nikolic T, Andric K, Petrovic D, Bolevich S et al (2022) Erythropoietin resistance development in hemodialysis patients: the role of oxidative stress. Oxid Med Cell Longev 2022:9598211 35464768 10.1155/2022/9598211 PMC9023176

[36] Lu X, Zhang J, Wang S, Yu Q, Li H (2020) High erythropoiesis resistance index is a significant predictor of cardiovascular and all-cause mortality in Chinese maintenance hemodialysis patients. Mediators Inflamm 2020:1027230 33293895 10.1155/2020/1027230 PMC7714563

[37] Neuen BL, Leather N, Greenwood AM, Gunnarsson R, Cho Y, Mantha ML (2016) Neutrophil–lymphocyte ratio predicts cardiovascular and all-cause mortality in hemodialysis patients. Ren Fail 38:70–76 26540580 10.3109/0886022X.2015.1104990

[38] Maeda A, Tsuruya K, Maeda M, Yamasaki M, Nakashima A, Nakashima Y et al (2019) Hemodialysis treatment time versus erythropoietin dose requirement: Reduction in 2,000 units per week by extension of hemodialysis for 1 hour. Clin Nephrol 92:174–179 31272526 10.5414/CN109403

