# Supplementary material for: Erythropoietin resistance among pediatric patients on chronic hemodialysis: A cross-sectional study
Source: Pediatr Nephrol. 2025 Apr 28;40(12):3725–32. doi: 10.1007/s00467-025-06776-4 (PMC12549759; doi:10.1007/s00467-025-06776-4)
Supplement: Supplementary file 1 — Graphical abstract (PPTX 36 KB) [file 467_2025_6776_MOESM1_ESM.pptx]

## Slide 1
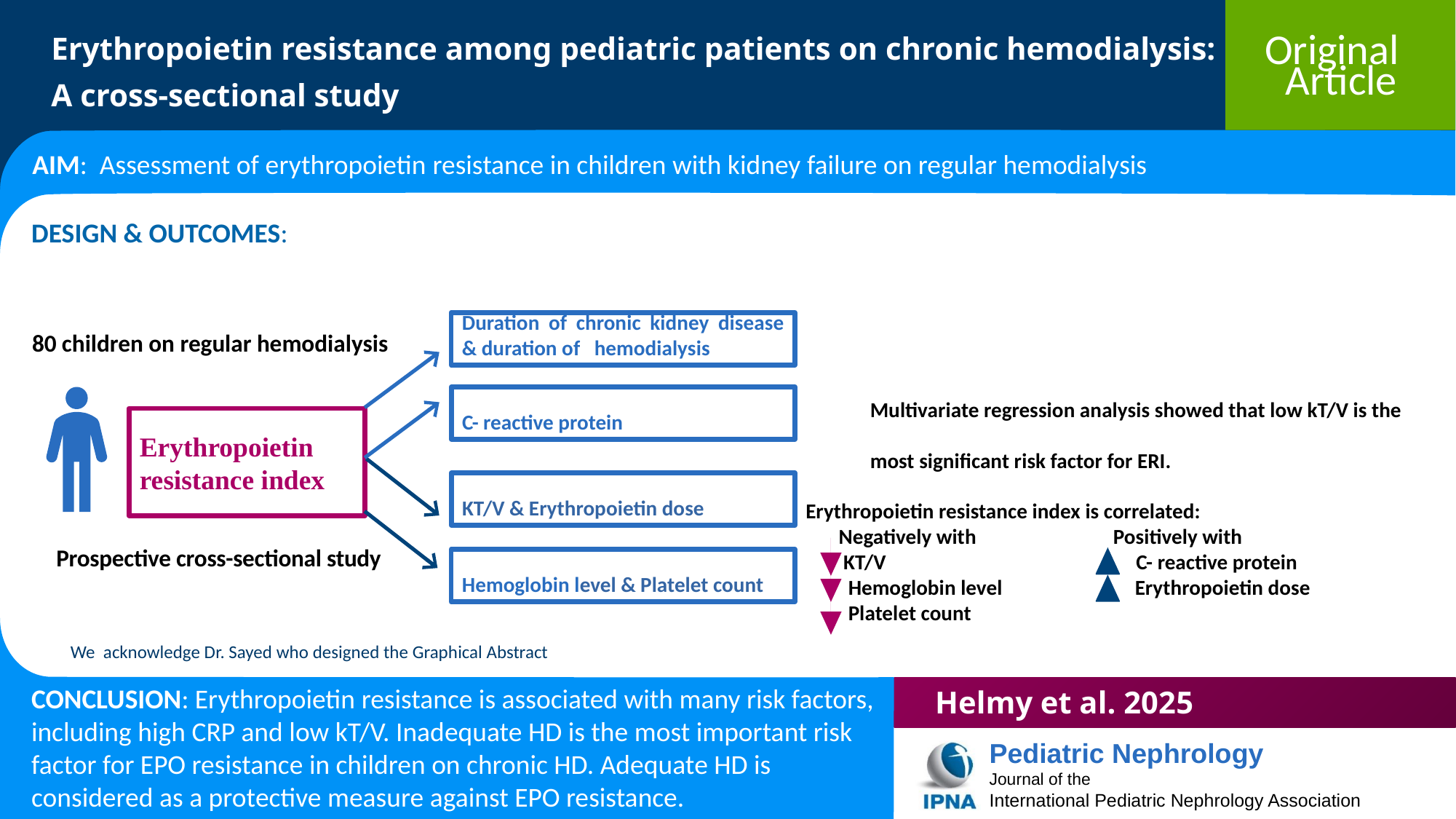

Erythropoietin resistance among pediatric patients on chronic hemodialysis:
A cross-sectional study
AIM: Assessment of erythropoietin resistance in children with kidney failure on regular hemodialysis
DESIGN & OUTCOMES:
Duration of chronic kidney disease & duration of hemodialysis
80 children on regular hemodialysis
Multivariate regression analysis showed that low kT/V is the most significant risk factor for ERI.
C- reactive protein
Erythropoietin resistance index
KT/V & Erythropoietin dose
Erythropoietin resistance index is correlated:
 Negatively with Positively with
 KT/V C- reactive protein
 Hemoglobin level Erythropoietin dose
 Platelet count
Prospective cross-sectional study
Hemoglobin level & Platelet count
We acknowledge Dr. Sayed who designed the Graphical Abstract
CONCLUSION: Erythropoietin resistance is associated with many risk factors, including high CRP and low kT/V. Inadequate HD is the most important risk factor for EPO resistance in children on chronic HD. Adequate HD is considered as a protective measure against EPO resistance.
 Helmy et al. 2025
