# Supplementary material for: Erythropoietin resistance among pediatric patients on chronic hemodialysis: A cross-sectional study
Source: Pediatr Nephrol. 2025 Apr 28;40(12):3725–32. doi: 10.1007/s00467-025-06776-4 (PMC12549759; doi:10.1007/s00467-025-06776-4)
Supplement: Supplementary file 2 — Supplementary file2 (DOCX 16 KB) [file 467_2025_6776_MOESM2_ESM.docx]

**Supplementary Table 1.** [**Laboratory investigations of the study population**](https://orcid.org/0000-0002-9009-165X)

|  | [**Mean ±SD**](https://orcid.org/0000-0002-9009-165X) | [**Range**](https://orcid.org/0000-0002-9009-165X) |
| --- | --- | --- |
| **H**[**emoglobin(g/dL)**](https://orcid.org/0000-0002-9009-165X) | [10.44±2.00](https://orcid.org/0000-0002-9009-165X) | [(5.20-16.10)](https://orcid.org/0000-0002-9009-165X) |
| [**Total leucocyte count (10^/cmm)**](https://orcid.org/0000-0002-9009-165X) | [7.18±3.07](https://orcid.org/0000-0002-9009-165X) | [(2.40-18.60)](https://orcid.org/0000-0002-9009-165X) |
| [**Platelet count (10^/cmm)**](https://orcid.org/0000-0002-9009-165X) | [245.29±101.64](https://orcid.org/0000-0002-9009-165X) | [(52.00-626.00)](https://orcid.org/0000-0002-9009-165X) |
| [**Lymphocyte (%)**](https://orcid.org/0000-0002-9009-165X) | [33.31±11.9](https://orcid.org/0000-0002-9009-165X)2 | [(10.00-66.00)](https://orcid.org/0000-0002-9009-165X) |
| [**Neutrophil (%)**](https://orcid.org/0000-0002-9009-165X) | [48.14±13.](https://orcid.org/0000-0002-9009-165X)01 | [(3.00-79.00)](https://orcid.org/0000-0002-9009-165X) |
| **Neutrophil Lymphocyte Ratio** | 1.78± 1.27 | (0.15- 7.90) |
| [**C-reactive protein**](https://orcid.org/0000-0002-9009-165X) **(mg/L)** | 10.80±9.52 | (6.00-48.00) |
| [**Sodium**](https://orcid.org/0000-0002-9009-165X)  [**(mmol/L)**](https://orcid.org/0000-0002-9009-165X) | [141.94±5.25](https://orcid.org/0000-0002-9009-165X) | [(126.00-155.00)](https://orcid.org/0000-0002-9009-165X) |
| [**Potassium**](https://orcid.org/0000-0002-9009-165X) [**(mmol/L)**](https://orcid.org/0000-0002-9009-165X) | [4.99±0.86](https://orcid.org/0000-0002-9009-165X) | [(2.60-8.00)](https://orcid.org/0000-0002-9009-165X) |
| [**Iron (ug/dL)**](https://orcid.org/0000-0002-9009-165X) | [71.07±22.98](https://orcid.org/0000-0002-9009-165X) | [(17.00-146.00)](https://orcid.org/0000-0002-9009-165X) |
| [**Ferritin (ng/mL)**](https://orcid.org/0000-0002-9009-165X) | [805.93±578.95](https://orcid.org/0000-0002-9009-165X) | [(96.00-3507.00)](https://orcid.org/0000-0002-9009-165X) |
| [**Total calcium (mg/dl)**](https://orcid.org/0000-0002-9009-165X) | [9.42±0.90](https://orcid.org/0000-0002-9009-165X) | [(6.30-11.90)](https://orcid.org/0000-0002-9009-165X) |
| [**Phosphorus (mg/dl)**](https://orcid.org/0000-0002-9009-165X) | [5.95±2.09](https://orcid.org/0000-0002-9009-165X) | [(2.10-10.90)](https://orcid.org/0000-0002-9009-165X) |
| **P**[**arathyroid hormone**](https://orcid.org/0000-0002-9009-165X) [**(pg/mL)**](https://orcid.org/0000-0002-9009-165X) | [156.36±240.62](https://orcid.org/0000-0002-9009-165X) | [(13.80-1523.00)](https://orcid.org/0000-0002-9009-165X) |
| [**Alkaline phosphatse. (U/L)**](https://orcid.org/0000-0002-9009-165X) | [279.45±186.60](https://orcid.org/0000-0002-9009-165X) | [(15.00-890.00)](https://orcid.org/0000-0002-9009-165X) |

[SD, standard deviation](https://orcid.org/0000-0002-9009-165X)
